# Efficacy and safety of Kanglaite injection combined with chemotherapy for colorectal cancer: A protocol for systematic review and meta-analysis

**DOI:** 10.1097/MD.0000000000022357

**Published:** 2020-09-25

**Authors:** Weili Mao, Yihua Fan, Chao Cheng, Xingyu Yuan, Tian Lan, Kaili Mao, Jun Wang

**Affiliations:** a People's Hospital of QuZhou, Quzhou, Zhejiang province; b First Teaching Hospital of Tianjin University of Traditional Chinese Medicine; c Tianjin University of Traditional Chinese Medicine, Tianjin, China.

**Keywords:** chemotherapy, colorectal cancer, Kanglaite injection, protocol, systematic review

## Abstract

**Background:**

The incidence and mortality of colorectal cancer are high. Chemotherapy is currently the commonly used therapeutic scheme, but there are drug resistance and toxic and side effects. Kanglaite (KLT) injection is a broad-spectrum anticancer drug extracted from *Semen Coicis (Yi Yi Ren)*, which has been widely used in the treatment of colorectal cancer. Clinical practice shows that KLT injection combined with chemotherapy has certain therapeutic advantages, but there is a lacking of evidence of evidence-based medicine. The purpose of this study is to systematically investigate the efficacy and safety of KLT injection combined with chemotherapy in the treatment of colorectal cancer.

**Methods:**

Randomized controlled trials of KLT injection combined with chemotherapy in the treatment of colorectal cancer were retrieved from English databases (PubMed, Embase, Web of Science, the Cochrane Library) and Chinese databases (China National Knowledge Infrastructure, Wanfang, Chongqing VIP Chinese Science and Technology Periodical Database, Chinese Biological and Medical database), as well as searching Baidu academic and Google academic manually, and the retrieval time was from their establishment to August 2020. Two researchers independently conducted data extraction and literature quality evaluation on the quality of the included literatures, and meta-analysis was conducted on the included literatures using RevMan 5.3 (developed by the UK's International Cochrane Collaboration).

**Results:**

This study assessed the efficacy and safety of KLT injection combined with chemotherapy in the treatment of colorectal cancer by effective rate, Karnofsky Performance Status, Carcinoemybryonic Angtigen remission rate, pain remission rate, and incidence of adverse reactions etc.

**Conclusions:**

This study will provide reliable evidence-based evidence for the clinical application of KLT injection combined with chemotherapy in the treatment of colorectal cancer.

**Ethics and dissemination:**

The private information from individuals will not be published. This systematic review also will not involve endangering participant rights. Ethical approval is not required. The results may be published in a peer-reviewed journal or disseminated in relevant conferences.

**OSF Registration number:**

DOI 10.17605/OSF.IO/EKVAF

## 1 Introduction

Colorectal cancer refers to a cancer occurring in the colon or rectum, which may occur in any part of the colorectal, but the rectum and sigmoid are the most common, it is a common and malignant tumor in the gastrointestinal tract. And it has high morbidity which is the third and mortality rate which is the second^[1–3]^ among common and malignant tumors in the gastrointestinal tract globally, while its morbidity is the fourth in China.^[4]^ Globally, there are 800,000 new cases of colorectal cancer every year, accounting for about 10% of global malignant tumors.^[5]^ The incidence of colorectal cancer is increasing year by year. Some studies have reported that the number of colorectal cancer patients will increase by more than 60% by 2030.^[6]^ Its pathogenesis has not been fully confirmed, but it is generally believed to be related to genetic factors, gene mutation and high-fat diet.^[7,8]^ The early symptoms of colorectal cancer are not obvious, but with the increase of cancers’ size, the symptoms such as hematochezia, diarrhea, change of bowel habits, local abdominal pain, anemia, and so on appear. The early symptoms of most patients are not easily detected. In the past 20 years, about 20% of patients had reached the advanced stage when clinical diagnosed and lost the best opportunity for treatment.^[9]^ At present, the treatment of advanced colorectal cancer is mainly based on chemotherapy, which can improve the survival rate and prolong the survival period of patients to a certain extent.^[10]^ However, patients” immune function will be affected to different extent in the long term chemotherapy, and there are short-term or long-term toxic and side effects.^[11]^ Therefore, it is necessary to seek alternative therapies with high efficacy and low side effects.

Chinese medicine is being widely used in the treatment of colorectal cancer.^[12,13]^ Clinical evidence shows that Chinese medicine has multi-targets, can be applied to multiple stages of colorectal cancer, and has reliable anti-inflammatory effects.^[14]^ KLT injection is a Chinese materia medical injection with anti-tumor activity.^[15]^ In 1997, KLT injection was officially approved by the Ministry of Public Health in China for the treatment of liver cancer, lung cancer, and gastric cancer.^[16,17]^ KLT has showed good efficacy in the USA and its clinical trial smoothly completed in Russia, where it has been registered as a new drug and can be marketed legally.^[18]^ At present, KLT injection is widely used in the treatment of cancers such as lung cancer, liver cancer, pancreatic cancer, gastric cancer, and colorectal cancer, with reliable clinical efficacy.^[19–23]^

Although a number of randomized controlled trials (RCTs) have shown that KLT injection combined with chemotherapy has the characteristics of enhancing the efficacy of chemotherapy and reducing the toxic and side effects in the treatment of colorectal cancer,^[24–27]^ there are differences in research protocols and efficacy among various clinical trials, which have affected the promotion of this therapy to some degree. Therefore, this study plans to systematically evaluate the effectiveness and safety of KLT injection combined with chemotherapy in the treatment of colorectal cancer, so as to provide reliable evidence-based proof for the clinical application of KLT injection in the treatment of colorectal cancer.

## 2 Methods

### 2.1 Protocol register

This protocol of systematic review and meta-analysis has been drafted under the guidance of the preferred reporting items for systematic reviews and meta-analyses protocols. Moreover, it has been registered on open science framework (OSF) on August 25, 2020. (Registration number: DOI 10.17605/OSF.IO/EKVAF).

### 2.2 Ethics

Since this is a protocol with no patient recruitment and personal information collection, the approval of the ethics committee is not required.

### 2.3 Eligibility criteria

#### 2.3.1 Types of studies

We will collected all available RCTs on KLT injection combined with chemotherapy for colorectal cancer, regardless of blinding, publication status, region, but language will be restricted to Chinese and English.

#### 2.3.2 Research objects

Patients with colorectal cancer who were definitely diagnosed, regardless of nationality, race, age, gender, or course of disease. Patients with other malignancies and non-primary colorectal cancer were excluded.

#### 2.3.3 Intervention measures

The treatment group was treated with KLT injection combined with chemotherapy. The control group was treated with the same chemotherapy regimen. (There is no limit on the dose and course of KLT injection, nor on the dose, frequency and course of chemotherapy.)

#### 2.3.4 Outcome indicators

(1) Primary outcome: ① the overall effective rate; ② Karnofsky Performance Status
(2) Secondary outcomes: ① Carcinoemybryonic Angtigen remission rate; ② pain remission rate; ③ incidence of adverse reactions.

### 2.4 Exclusion criteria

(1) Literatures published repeatedly;
(2) Literatures which are abstracts or have incomplete data and whose data cannot be obtained after contacting the author;
(3) Literatures with obvious data errors;
(4) Literature with high bias risk by randomization or allocation concealment assessment^[28]^;
(5) Literatures in which the intervention measures are inconsistent with the literatures;
(6) Literatures with no relevant outcome indicators.

### 2.5 Search strategy

“Kang Lai Te (Kanglaite)”, “Jie Zhi Chang Ai (colorectal cancer)”, “Jie Zhi Chang Ai Zhong (colorectal neoplasm)”, “Zhi Chang Ai (rectal cancer)” and “Da Chang Ai (colorectal cancer)” were searched in Chinese databases, including China National Knowledge Infrastructure, Wanfang, Chongqing VIP Chinese Science, Technology Periodical Database and Chinese Biological and Medical database; “Kanglaite”, “KLT”, “Colorectal Neoplasm” and “Colorectal Carcinoma” etc were retrieved in English database, including PubMed, EMBASE, Web of Science, the Cochrane Library. In addition, manually searched on Baidu academic and Google academic. The retrieval time was from establishment of the databases to August 2020, and all domestic and foreign literatures on KLT injection combined with chemotherapy for colorectal cancer were collected. Take PubMed as an example, and the search strategy is shown in Table 1.

**Table 1:**
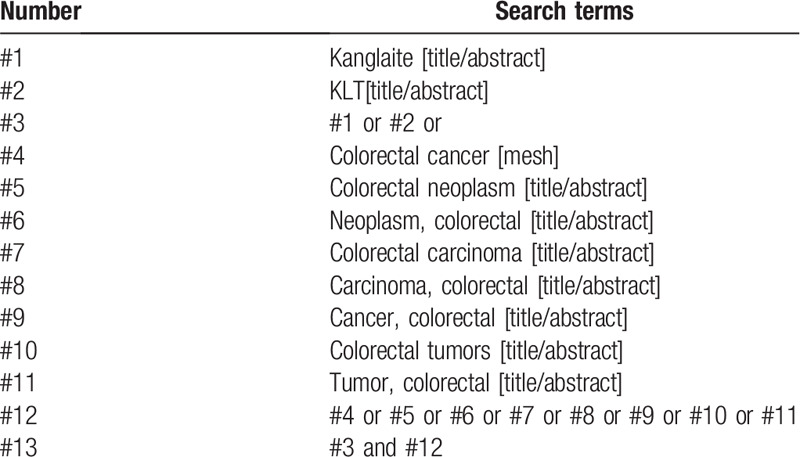
Search strategy in PubMed database.

### 2.6 Data screening and extraction

**Figure 1:**
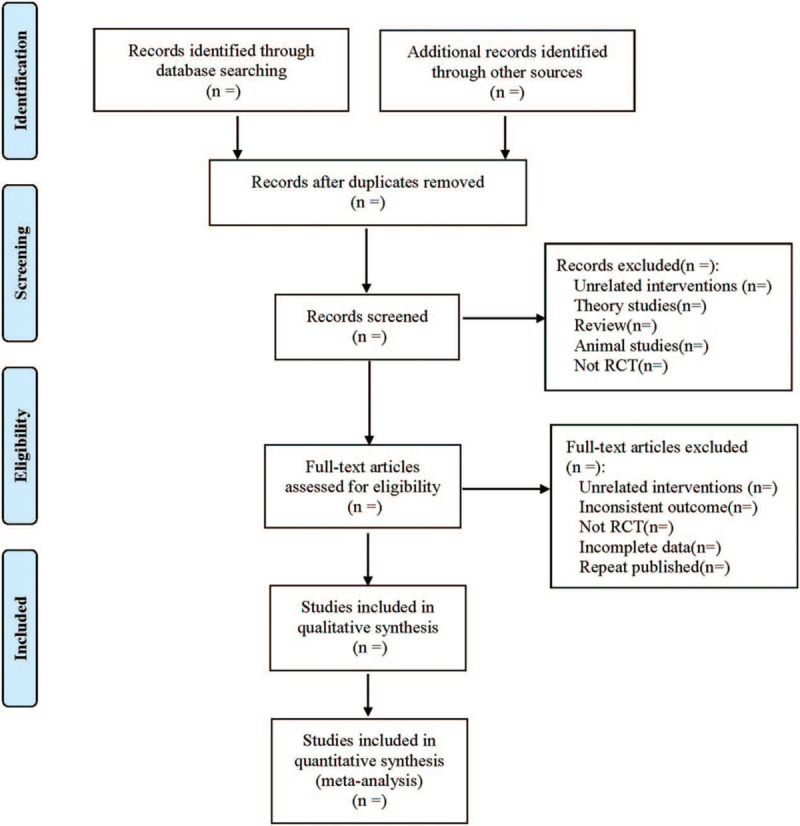
Flow diagram.

Referring to the method of research selection in version 5.0 of the Cochrane collaboration Network system Evaluator Manual, according to the the Preferred Reporting Items for Systematic Reviews and Meta-Analyses flow chart, the 2 researchers used the EndNote X9 document management software to independently screen and check the literature according to the above inclusion and exclusion criteria, and check each other, if there were different opinions, negotiate with a third party to resolve the differences. At the same time, Excel 2013 was used to extract relevant information, including: ① Clinical study (title, first author, date of publication, sample size, sex ratio, average age, average course of disease, tumor stage, and type); ② Intervention measures (dose, frequency and course of KLT injection in the treatment group; chemotherapy regimen, intensity, frequency and course of treatment for treatment group, and control group); ③ Risk bias assessment elements in RCTs; ④ Observation targets. The selection process of literature is shown in Figure 1.

### 2.7 Literature quality assessment

Use the Cochrane collaboration's tool for assessing risk of bias to do the risk of bias assessment of included studies. According to the performance of the included literatures in the above evaluation items, 2 researchers will give judgments like low risk, unclear, or high risk judgments one by one, and cross-check after completion respectively. In case of any disagreement, discussion will be carried out. If no agreement can be reached between the 2, discussion will be made with the researchers in the third party.

### 2.8 Statistical analysis

#### 2.8.1 Data analysis and processing

The RevMan 5.3 software (developed by the UK's International Cochrane Collaboration) provided by the Cochrane Collaboration will be used for statistical analysis. ① Relative risk is selected as the statistic for the dichotomous variable. For continuous variables, Weighted Mean Difference is selected when the tools and units of measurement indicators are the same, Standardized Mean Difference is selected with different tools or units of measurement, and all the above are represented by effect value and 95% Confidence interval. ② Heterogeneity test: Q test is used to qualitatively determine inter-study heterogeneity. If *P*≥.1, there is no inter-study heterogeneity; If *P* < .1, it indicate inter-study heterogeneity. At the same time, I^2^ value is used to quantitatively evaluate the inter-study heterogeneity. If I^2^≤50%, the heterogeneity is considered to be good, and the fixed-effect model is adopted. If I^2^ > 50%, it is considered to be significant heterogeneity, the source of heterogeneity will be explored through subgroup analysis or sensitivity analysis. If there is no obvious clinical or methodological heterogeneity, it will be considered as statistical heterogeneity, and the random-effect model will be used for analysis. Descriptive analysis will be used if there is significant clinical heterogeneity between the 2 groups and subgroup analysis is not available.

#### 2.8.2 Dealing with missing data

If there is missing data in the article, contact the author via email for additional information. If the author cannot be contacted, or the author has lost relevant data, descriptive analysis will be conducted instead of meta-analysis.

#### 2.8.3 Subgroup analysis

According to the age, patients were divided into young and old for subgroup analysis. Subgroup analysis was performed according to the stages of disease course. Subgroup analysis was performed according to the course of treatment. Subgroup analysis was performed according to different chemotherapy regimens.

#### 2.8.4 Sensitivity analysis

In order to test the stability of meta-analysis results of indicators, a one-by-one elimination method will be adopted for sensitivity analysis.

#### 2.8.5 Assessment of publication bias

Funnel plots were used to assess publication bias if no fewer than 10 studies were included in an outcome measure. Moreover, Egger and Begg test were used for the evaluation of potential publication bias.

#### 2.8.6 Evidence quality evaluation

The Grading of Recommendations Assessment, Development, and Evaluation will be used to assess the quality of evidence. It contains 5 domains (bias risk, consistency, directness, precision, and publication bias). And the quality of evidence will be rated as high, moderate, low, and very low.

## 3 Discussion

Colorectal cancer belongs to the category of diseases like “abdominal mass (Zheng Jia)”, “amassment and accumulation (Ji Ju)” and “intestinal wind (Chang Feng)”etc in traditional Chinese medicine.^[29]^ High incidence, poor prognosis and high mortality of colorectal cancer seriously harm people's health.^[30]^ At present, the treatment schemes include surgical treatment, radiotherapy, chemotherapy, targeted therapy, immunotherapy, and traditional Chinese medical treatment, etc. Due to the hidden of early symptoms, it is already in the middle and advanced stage when diagnosed, and mainly uses chemotherapy to treat. But chemotherapy often fails because of drug resistance and the intolerance of toxic and side effects of chemotherapy.

Kanglaite (KLT) injection is extracted from *Semen Coicis (Yi Yi Ren)*, and *Semen Coicis (Yi Yi Ren)* has anti-tumor and immunomodulatory effects confirmed by modern pharmacology,^[31,32]^ KLT principally blocks cell cycle at G2/M phase to reduce cellular mitotic division and inhibit proliferation of tumour cells. KLT can also activate pro-apoptotic factors and lead cells to apoptosis.^[33]^ It has been clinically verified that KLT injection can effectively reverse multidrug resistance of tumor cells and improve the sensitivity of tumor cells to chemotherapy.^[20,22,34]^ In recent years, KLT injection has been widely used in the treatment of colorectal cancer, with significant efficacy.^[35]^ Yet the evidence from the RCTs is inconsistent. With the increasing number of clinical trials, it is urgent to systematically evaluate the efficacy of KLT injection in the treatment of colorectal cancer. In this study, we will summarize the latest evidence on the efficacy of KLT injection combined with chemotherapy for colorectal cancer. This work also provides useful evidence to determine whether KLT injection is effective and safe to patients with colorectal cancer, which is beneficial to both clinical practice and health-related decision-makers.

However, this systematic review has some limitations. The types and doses of chemotherapy regiments used in the included studies were different, which may cause some clinical heterogeneity. There are differences in course of the diseases of patients that may influence the outcome. In addition, due to the limitation of language ability, we only search literatures in English and Chinese, and may ignore research or reports in other languages.

## Author contributions

**Data curation:** Weili Mao, Yihua Fan.

**Formal analysis:** Weili Mao, Yihua Fan.

**Funding acquisition:** Jun Wang.

**Funding support:** Jun Wang.

**Literature retrieval:** Chao Cheng and Xingyu Yuan.

**Resources:** Chao Cheng, Xingyu Xingyu Yuan.

**Software operating:** Tian Lan and Kaili Mao.

**Software:** Chao Cheng, Tian Lan, Kaili Mao.

**Supervision:** Jun Wang.

**Writing – original draft:** Weili Mao, Yihua Fan.

**Writing – review & editing:** Weili Mao and Jun Wang.
